# Classification of SLC family-related genes involved in ferroptosis predicts lung cancer prognosis and immunotherapy response

**DOI:** 10.1038/s41598-023-47328-w

**Published:** 2023-11-16

**Authors:** Shun Gao, Guotao Gong, Xinyi Wang, Xinrui Gao, Xuanzhu Guo, Yuyao Luo, Sijie Li, Yan Zhang, Sheng Lin

**Affiliations:** 1 Department of Oncology, The Affiliated Hospital of Southwest Medical University, Luzhou, Sichuan China; 2 Department of Oncology, Luzhou Municipal People’s Hospital, Luzhou, Sichuan China

**Keywords:** Cancer, Computational biology and bioinformatics, Genetics

## Abstract

Lung adenocarcinoma, the most frequent type of lung cancer, is the leading cause of cancer-related deaths worldwide. Ferroptosis, controlled cell death that involves a high degree of iron-dependent lipid peroxidation, has been linked to tumor therapy sensitivity, patient prognosis, and cancer development. The solute carrier superfamily has over 400 members and comprises the largest class of transporters in the human genome. Solute carrier proteins can facilitate the movement of different substrates across biological membranes, which is crucial for physiological activities, including ferroptosis. Here, we developed a new model to further explore the role of the solute carrier family in ferroptosis in the lung adenocarcinoma immunological milieu. We used consensus clustering to classify patients with lung cancer into two subgroups (cluster1 and cluster2). Patients in the cluster1 subtype had a better prognosis and higher immune cell infiltration ratios than those in the cluster2 subtype. Furthermore, to evaluate the prognosis, the immune cell infiltration ratio, and the medication sensitivity of patients with lung adenocarcinoma, we developed gene scores related to the solute carrier family. In conclusion, we successfully developed a model incorporating the solute carrier family and ferroptosis to predict survival and the impact of immunotherapy on patients with lung cancer.

With an extremely high incidence rate worldwide, lung cancer has now overtaken all other cancers as the greatest threat to human health^1^. Based on histological categorization, lung cancer can be divided into small cell lung cancer and non-small cell lung cancer. Non-small cell lung cancers include lung adenocarcinoma (LUAD), lung squamous cell carcinoma, lung adenosquamous cell carcinoma, and large cell lung cancer^2^. LUAD is the predominant histological type, and its prevalence continues to increase^3^. Regarding therapies, targeted immunotherapy has led to notable advances in the treatment of lung cancer in recent years^4^. However, the overall survival rates of patients with LUAD are poor, and these treatments are only effective in some patients^5^. Therefore, we must identify novel and accurate disease markers to effectively treat patients with LUAD. In addition, it is crucial to develop a more accurate prognostic model.

Ferroptosis is an iron-mediated method of cell death. Apoptosis, autophagy, necrosis, and scorch are morphologically related to this mechanism of cell death^6^. Recent studies have shown that ferroptosis is closely related to the pathophysiological mechanisms of various diseases, including cancer, diseases of the nervous system, kidney damage, ischemia-reperfusion injury, and blood disorders^7^. Inducing ferroptosis in cancer cells has emerged as a novel cancer treatment strategy in recent years, particularly for types of cancer that respond poorly to conventional treatments, including radiation, chemotherapy, and immunotherapy^8^. In addition, ferroptosis and tumor immunotherapy are strongly correlated^9^. Ferroptosis-related medications are gradually being used in clinics^10^; therefore, determining the function of ferroptosis regulators in tumor therapy is becoming increasingly crucial.

The solute carrier (SLC) family, second only to G-protein-coupled receivers, which rank first in number^11^, are essential components of the cell and organelle membranes^12^. In addition, proteins from the SLC family play a critical role in the physiology and transport of a wide range of molecules, including waste removal, nutrient absorption, ion transport, and medication absorption and disposal^13^. Furthermore, SLC family proteins are crucial for ferroptosis and influence the tumor microenvironment^14,15^. However, the role of SLC genes in the regulation of ferroptosis in patients with cancer and how it affects patient response to immunotherapy have not yet been investigated. To predict the outcome of patients with LUAD and the impact of immunotherapy, we created a set of genotyping models for SLC family-related genes (SFRGs) in ferroptosis. Furthermore, our objective was to develop an SFRG risk score to predict the survival outcome, impact of immunotherapy, and medication sensitivity in patients with LUAD.

## Results

### Consensus clustering identifies two subtypes of SFRGs

**Figure 1:**
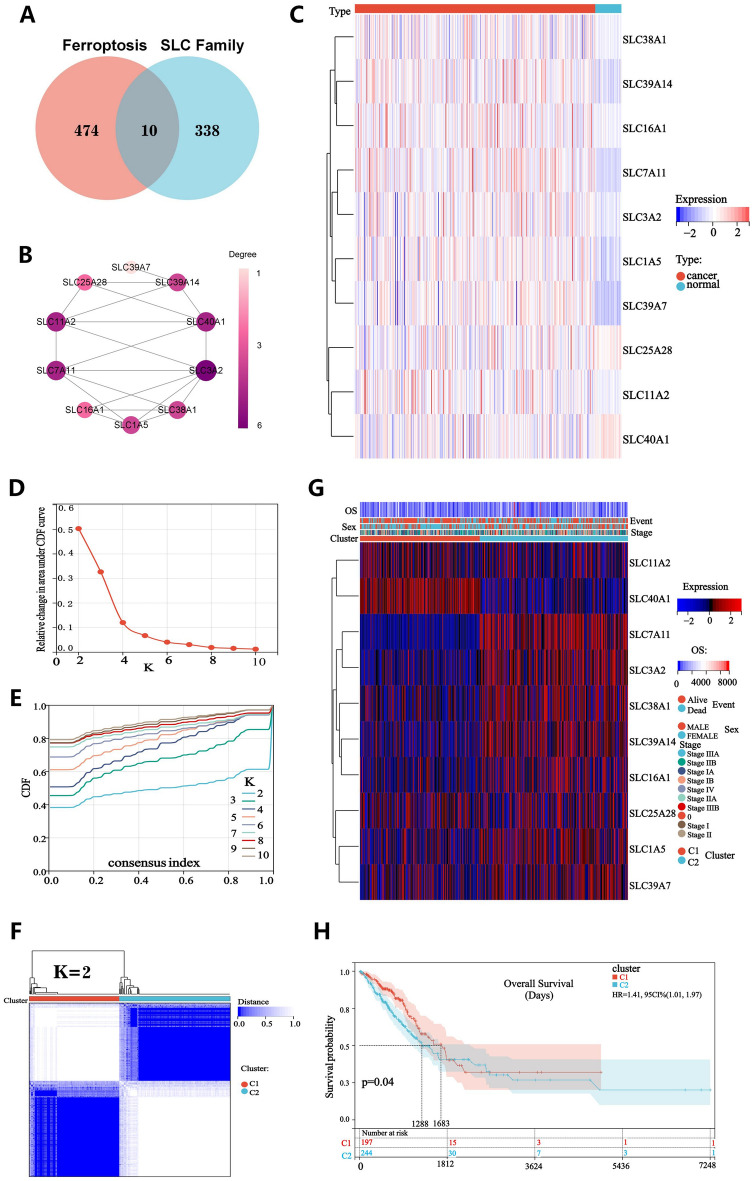
Consensus clustering identifies lung adenocarcinoma subtypes associated with SFRGs. (**A**) Ten SFRGs were identified. (**B**) Protein–protein interactions between SFRGs. (**C**) The gene expression profiles of 10 SFRGs in normal tissues and lung adenocarcinoma samples from the TCGA cohort are depicted in a heatmap. (**D**) Sample clustering consistency, area under the distribution curve. (**E**) The cumulative distribution curve from k = 2 to k = 10 in consensus clustering are all columnar. (**F**) Consensus clustering heatmap at k = 2. (**G**) Heatmap of SFRG expression in the two different subtypes. (**H**) Kaplan–Meier curves of OS in cluster1 and cluster2 subtypes. SFRGs, SLC family-related genes; OS, overall survival; TCGA, The Cancer Genome Atlas.

We obtained the SLC family genes in the Genecards database, and the ferroptosis genes in the FerrDB database were intersected to obtain the SRFGs genes list (Fig. 1A). We imported 10 SFRGs genes into the string(https://cn.string-db.org/) database to obtain the node information of their interaction networks. Then, we imported this information into Cytoscape software for analysis or Degree scoring (Supplementary data), and plotted the SFRGs genes according to the Degree scoring (Fig. 1B). A paired sample test was used to compare the differential expression of SFRGs between normal and paired tumor tissues (Fig. 1C). With a maximum clustering of k = 2, we assessed the samples based on the area under the cumulative distribution function (CDF) curve and average consistency within the cluster group (Fig. 1D,E). The cohort patients were classified into two groups, C1 (n = 203) and C2 (n = 250), based on 453 patients with LUAD in The Cancer Genome Atlas (TCGA) database (Fig. 1F). The prognosis of patients with the C1 subtype was better than that of patients with the C2 subtype, as determined using a Kaplan–Meier survival calculation. After creating a heatmap showing the expression relationship of SFRGs, we discovered that most SFRGs were highly expressed in the C2 group (Fig. 1G). Finally, we evaluated the significant differences in prognosis among various groups of samples using the log-rank test (Fig. 1H).

### Form for clinical information

**Figure 2:**
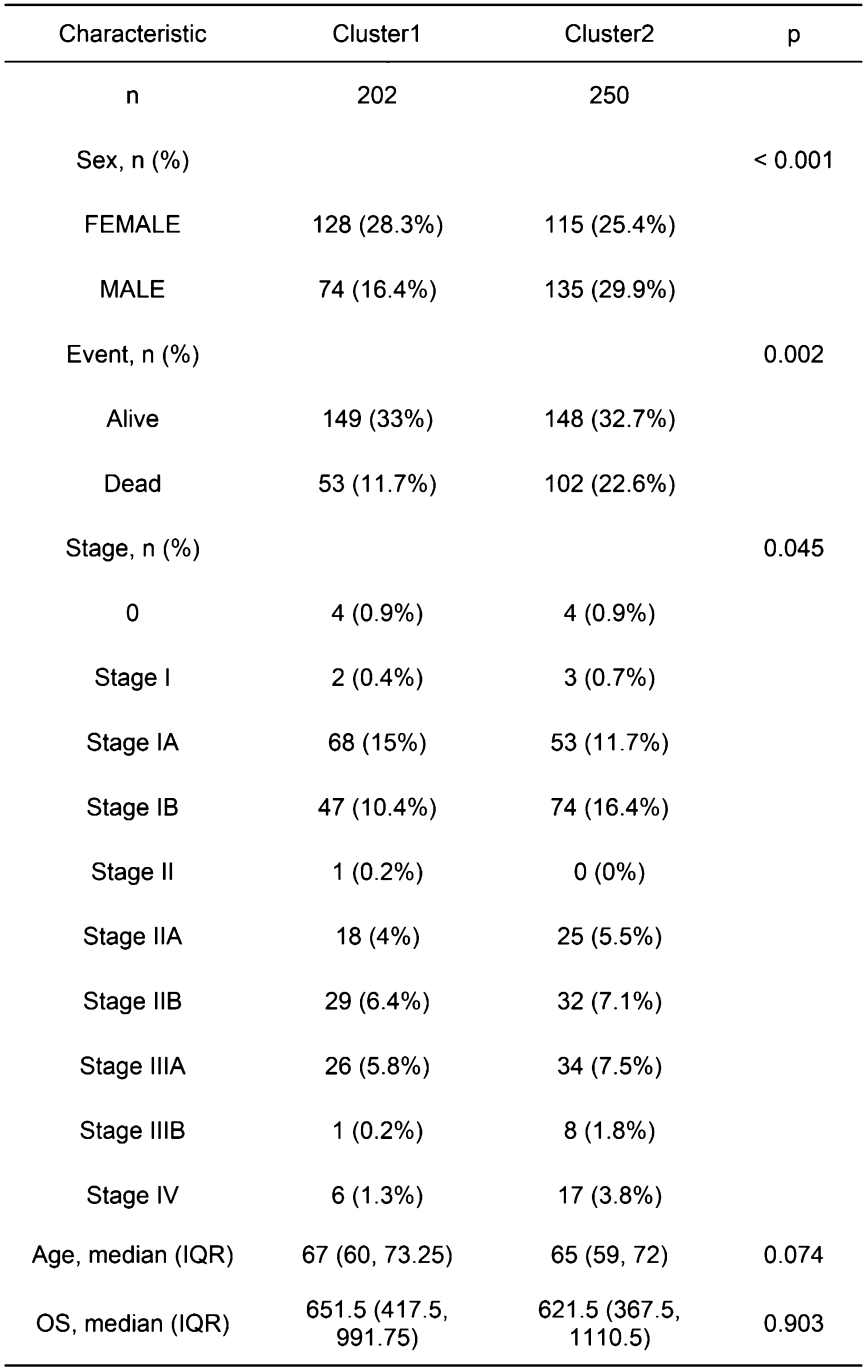
Form for clinical information.

Through chi square test analysis of clinical data related to patients with Cluster1 and Cluster2 subtypes, we found that the proportion of male patients with C2 subtype was higher than that with C1 subtype, and the death outcome of C2 subtype patients was significantly higher than that of C1 subtype patients. Finally, the clinical staging of C2 subtype is mainly in stage IB, while the staging of C1 subtype is mainly in stage IA. In addition, the proportion of C2 subtype patients in stage IIA, stage IIB, stage IIIA, stage IIIB, and stage IV staging is higher than that of C1 subtype patients (Fig. 2).

### Identification of Differentially Expressed Genes (DEGs) and Signaling Pathways in Patients with Subtypes C1 and C2

**Figure 3:**
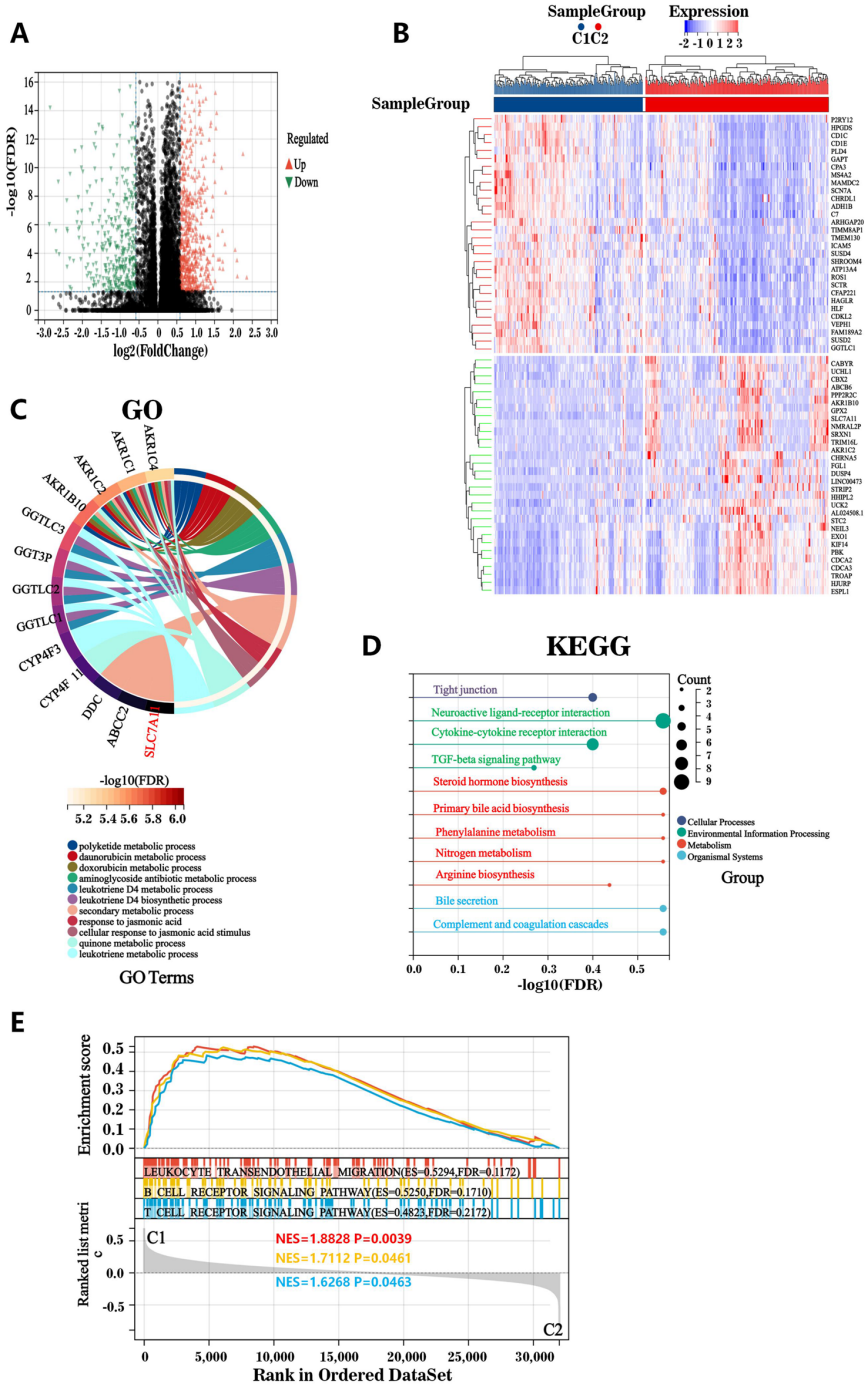
Differential gene expression and potential signaling pathways in different patient subtypes. (**A**) Volcano plot quantifying the differentially expressed genes between subtypes C1 and C2. (**B**) Heatmap showing the expression relationship of the top 40 differential genes between the C1 and C2 subtypes. (**C** and **D**) Enrichment analysis of the GO and KEGG pathways. (**E**) GSEA of the potential immune signaling pathways between patients with the C1 and C2 subtypes.

Using the R software t-test function, 1,003 DEGs were detected between patients with the subtypes cluster1 and cluster2 (Fig. 3A,B). We then performed functional enrichment analyses on these DEGs using the Gene Ontology (GO) and Kyoto Encyclopedia of Genes and Genomes (KEGG) databases. The DEGs were associated with the immunological microenvironment, signaling, material movement, cell development, and death (Fig. 3C,D). Therefore, the two SFRG subtypes had a unique relationship with the immunological microenvironment. Using Gene Set Enrichment Analysis (GSEA), we showed that several immune route-related pathways, including the B-cell pathway, the T-cell pathway, and leukocyte transendothelial migration, were differentially enriched in the two SFRG subtypes (Fig. 3E).

### Somatic mutation landscape of the C1 and C2 subtypes

**Figure 4:**
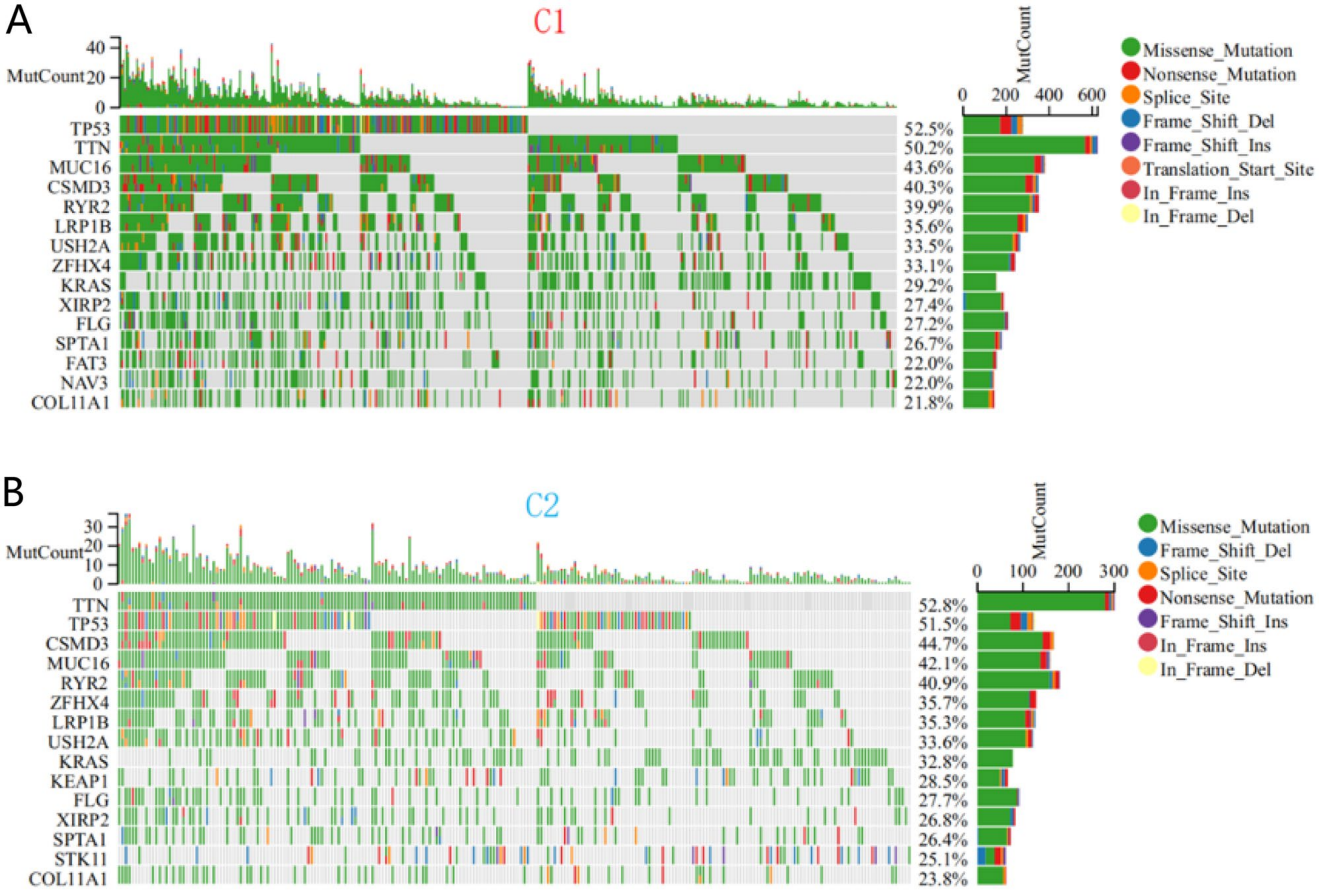
Comparison of somatic mutations and SFRGs between different subtypes. Top 15 frequently mutated genes in patients with (**A**) subtypes C1 and (**B**) subtypes C2.

When comparing patients with C1 and C2 subtypes, the most common mutations in the C1 subtype are FAT3 (22.0%) and NAV3 (22.0%), while the C2 subtype is KEAP1 (28.5%) and STK11 (25.1%), In existing research reports, compared with non mutated LUAD patients, LUAD patients with FAT3 mutations have significantly longer immunotherapy progression free survival (PFS)^16^. In addition, among non-small cell lung cancer patients, KEAP1 and STK11 mutated patients are not sensitive to immunotherapy and have shorter disease-free and overall survival^17,18^ (Fig. 4).

### Mutational landscape of patients with subtypes C1 and C2 in the tumor microenvironment

**Figure 5:**
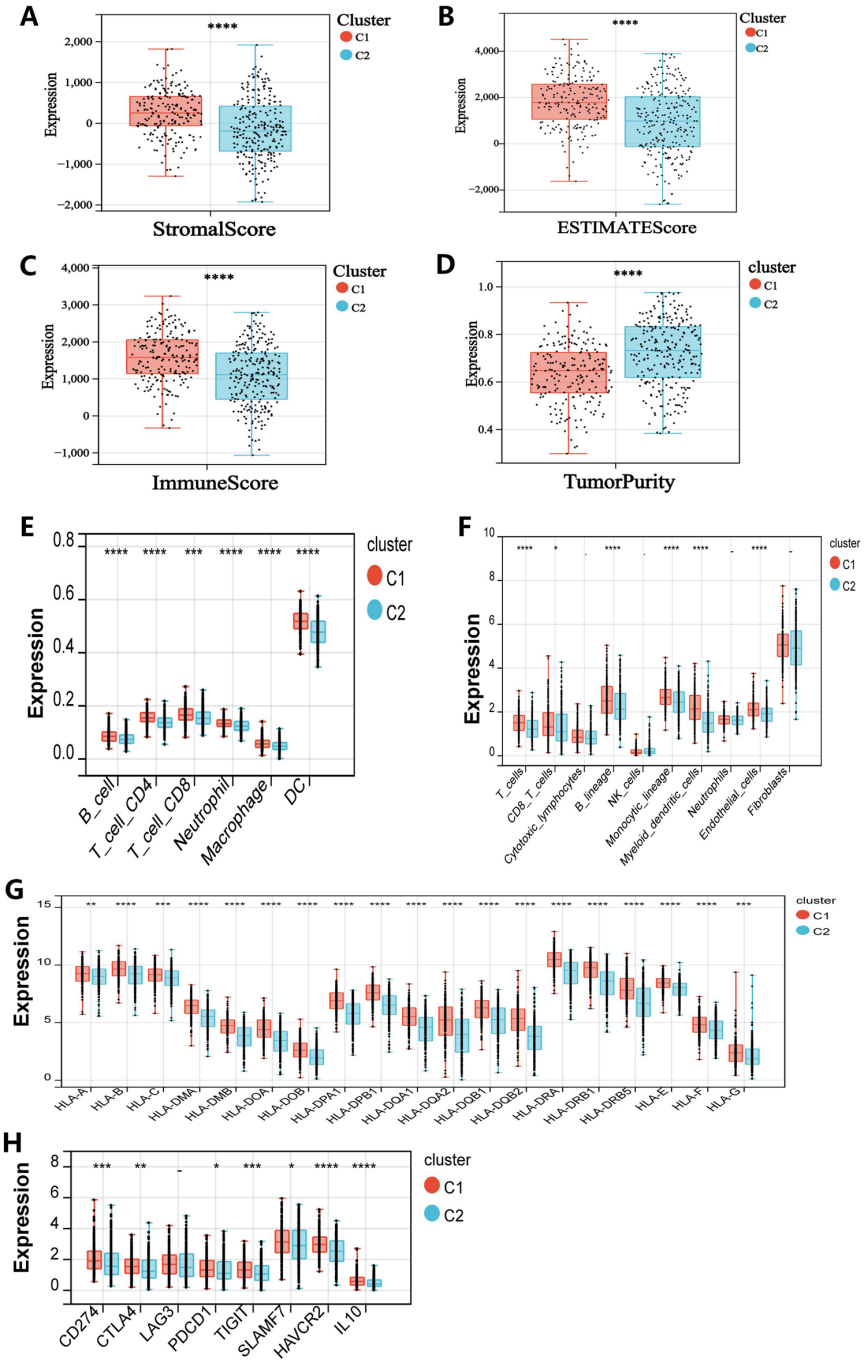
Immune landscape of patients with subtypes C1 and C2. (**A**–**D**) Small ladder diagram comparing patients with C1 and C2 subtypes regarding the three immune scores and tumor purity. (**E**) and (**F**) show significantly different immune cell infiltration between the different subtypes. (**G**) and (**H**) show significant differences in immune checkpoints and HLA cells between patients with different subtypes. “**-**” = ns; *P < 0.05; **P < 0.01; ***P < 0.001; ****P < 0.0001.

To better understand the impact of SFRGs typing on immune cell infiltration in LUAD, we examined the differences in the tumor microenvironment between the C1 and C2 subtypes. Overall, the C1 subtype had a higher StromalScore, ESTIMATEScore, and ImmunueScore, and lower TumorPurity than the C2 subtype (Fig. 5A–D). The immune cell infiltration ratio was then examined using MCPcounter and TIMER. Patients with subtype C1 had showed higher percentages of immune cell infiltration than those with subtype C2 (Fig. 5E,F). Furthermore, individuals with subtype C1 had higher rates of immune checkpoints and human leukocyte antigen (HLA) cell infiltration than patients with subtype C2 (Fig. 5G,H).

### SFRG analysis using univariate regression analysis

**Figure 6:**
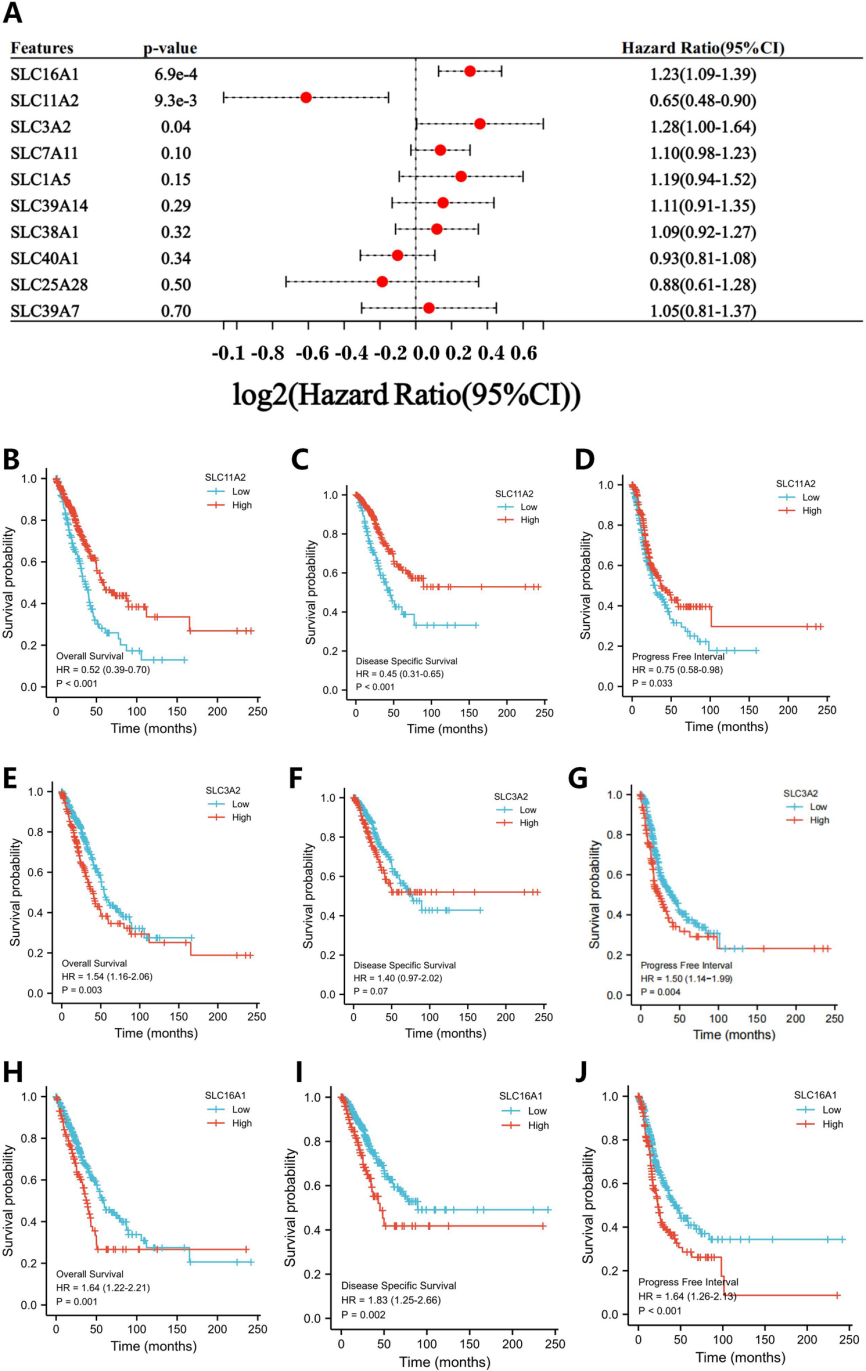
Univariate regression analysis. (**A**) Analysis of 10 SFRGs using a univariate regression analysis. (**B**–**J**) The OS, DSS, and PFI of SLC11A2, SLC3A2, and SLC16A1. SFRGs, SLC family-related genes; OS, overall survival; DSS, disease specific survival; PFI, progress free interval.

We performed a univariate regression analysis to identify three independent prognostic indicators that could be used in lung adenocarcinoma patients (Fig. 6A). Patients with high SLC11A2 expression have greater overall survival (OS), DSS, and progress free interval (PFI) scores than patients with low SLC11A2 expression. In contrast, patients with high SLC3A2 expression have lower OS, DSS, and PFI scores than patients with low SLC3A2 scores, whereas DSS is unaffected (Fig. 6B–J).

### Construction and validation of gene risk models related to SFRGs

**Figure 7:**
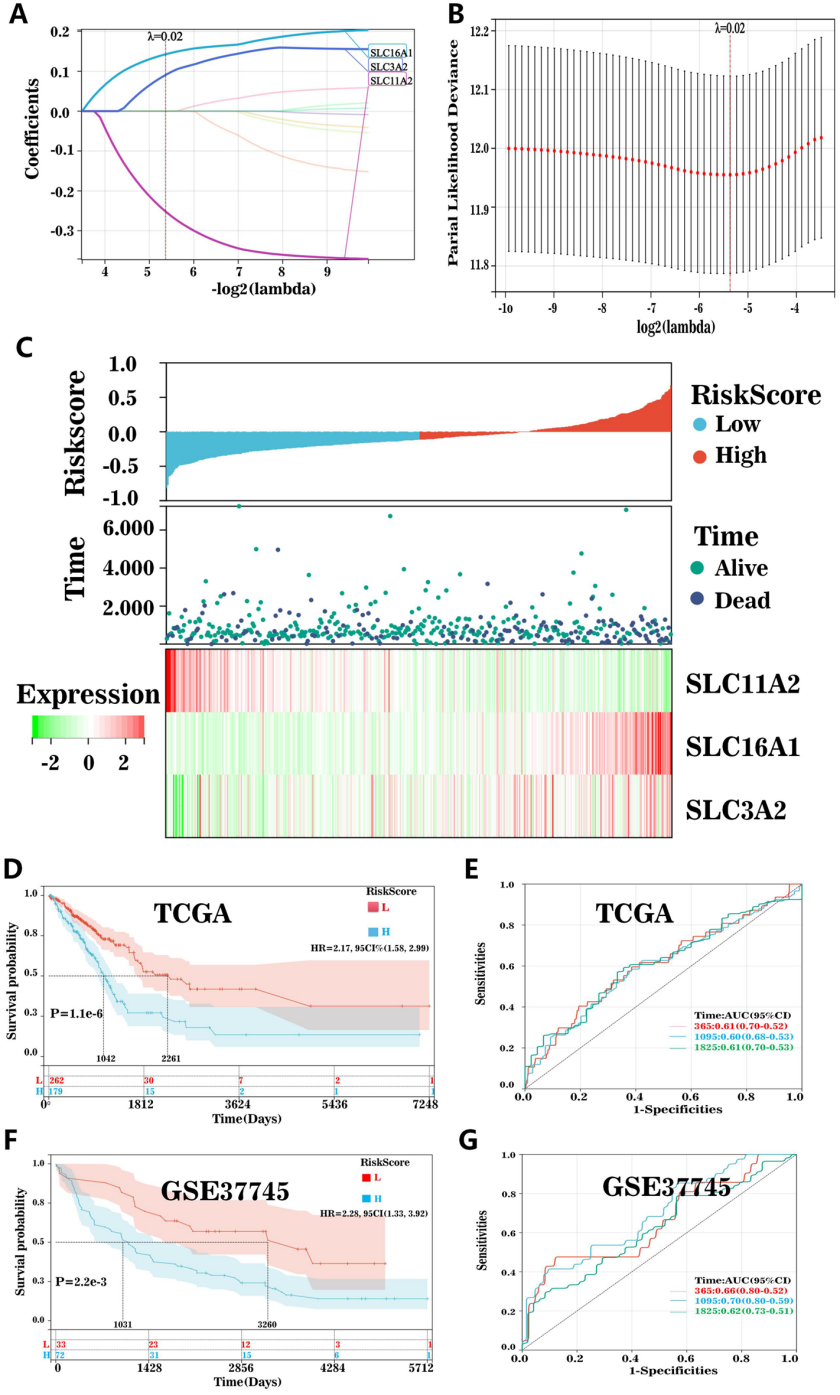
Construction and validation of risk scores. (**A**) and (**B**) Lasso regression identified three SFRGs most associated with OS in the TCGA dataset. (**C**) Characteristic heatmaps of risk score distribution, prognostic status, and three prognostic genes for each patient in TCGA database. Kaplan–Meier analysis demonstrating the prognostic significance of the risk model in (**D**–**G**) TCGA and (**D**) GSE37745 cohorts. SFRGs, SLC family-related genes; OS, overall survival; TCGA, The Cancer Genome Atlas.

We combined the data on gene expression, survival time, and survival status and used the Lasso–Cox method for regression analysis; consequently, three genes were obtained for the prediction model (Fig. 7A,B). The association between the risk of these three genes and survival status was also investigated. The survival rate of patients remarkably declined as the number of risk variables increased. As anticipated, SLC16A1, SLC3A2, and SLC11A2 were risk factors (Fig. 7C). In the TCGA cohort, patient prognoses were negatively correlated with lung cancer risk scores (Fig. 7D,E). We then verified this finding in a GEO cohort study (Fig. 7F,G).

### Correlation of the risk characteristics of SFRGs with subtypes c1 and c2 and the tumor microenvironment

**Figure 8:**
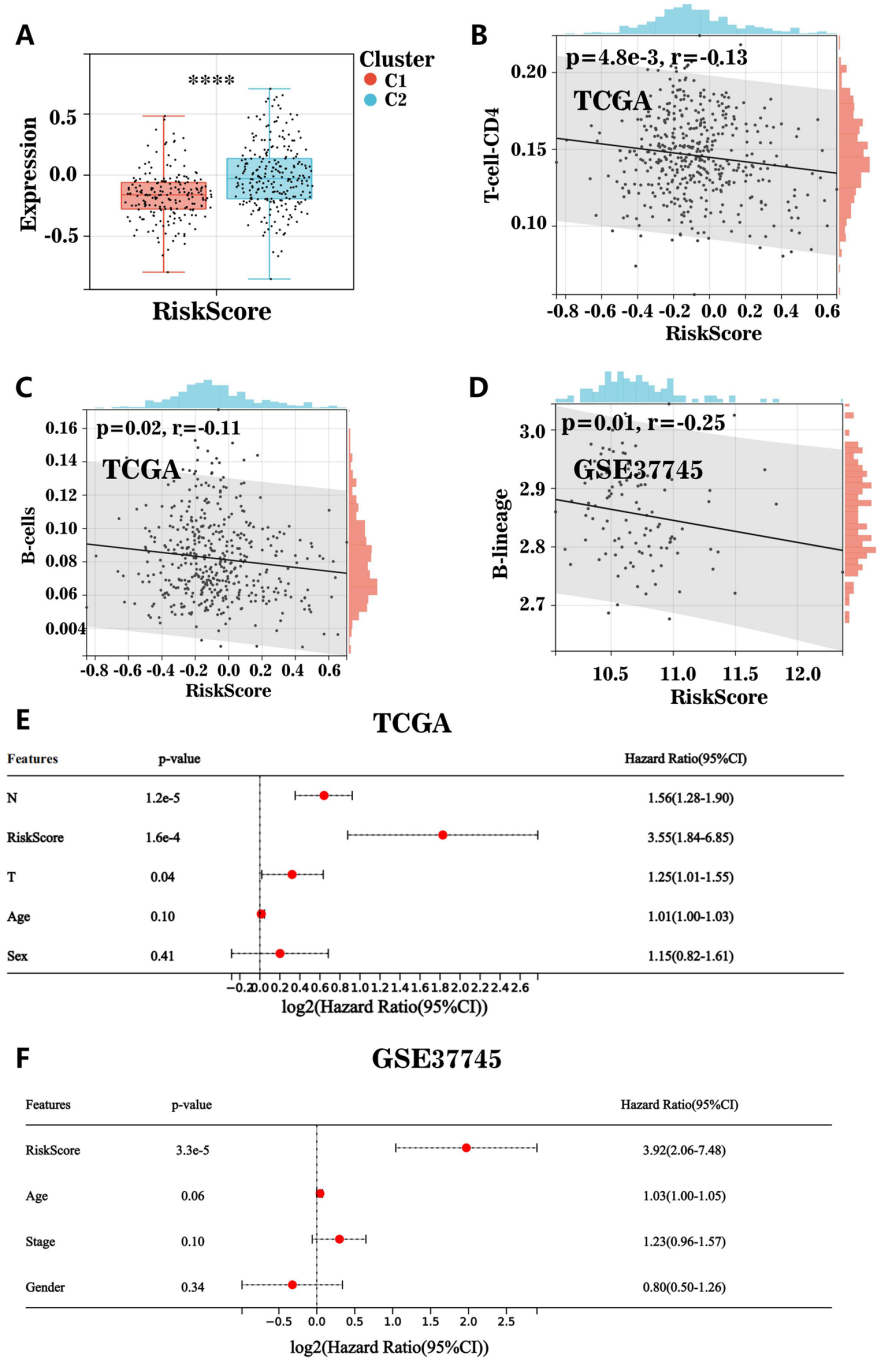
Relationship between the risk characteristics of SFRGs and the tumor microenvironment. (**A**) Relationship between risk scores among patients with different subtypes. (**B**–**D**) Scatter plot showing the correlation between risk score and immune cells (B cells and CD4 T cells). (**E**) and (**F**) Multivariate Cox regression analysis that evaluates the independent prognostic value of SFRG risk factors in patients with lung adenocarcinoma. SFRGs, SLC family-related genes.

Patients with subtype C2 had higher risk scores than those with subtype C1 (Fig. 8A). We also examined the association between the tumor microenvironment and the SFRG risk score. The risk score of SFRGs was adversely associated with B-cell and CD4 T-cell infiltration (Fig. 8B–D). These findings were validated in the GEO cohort. To assess the independent predictive value of SFRG risk factors, a multivariate Cox analysis was used to determine the risk variables of SFRGs as independent prognostic factors for patients with LUAD (Fig. 8E,F).

### Multivariate nomograms to predict survival

**Figure 9:**
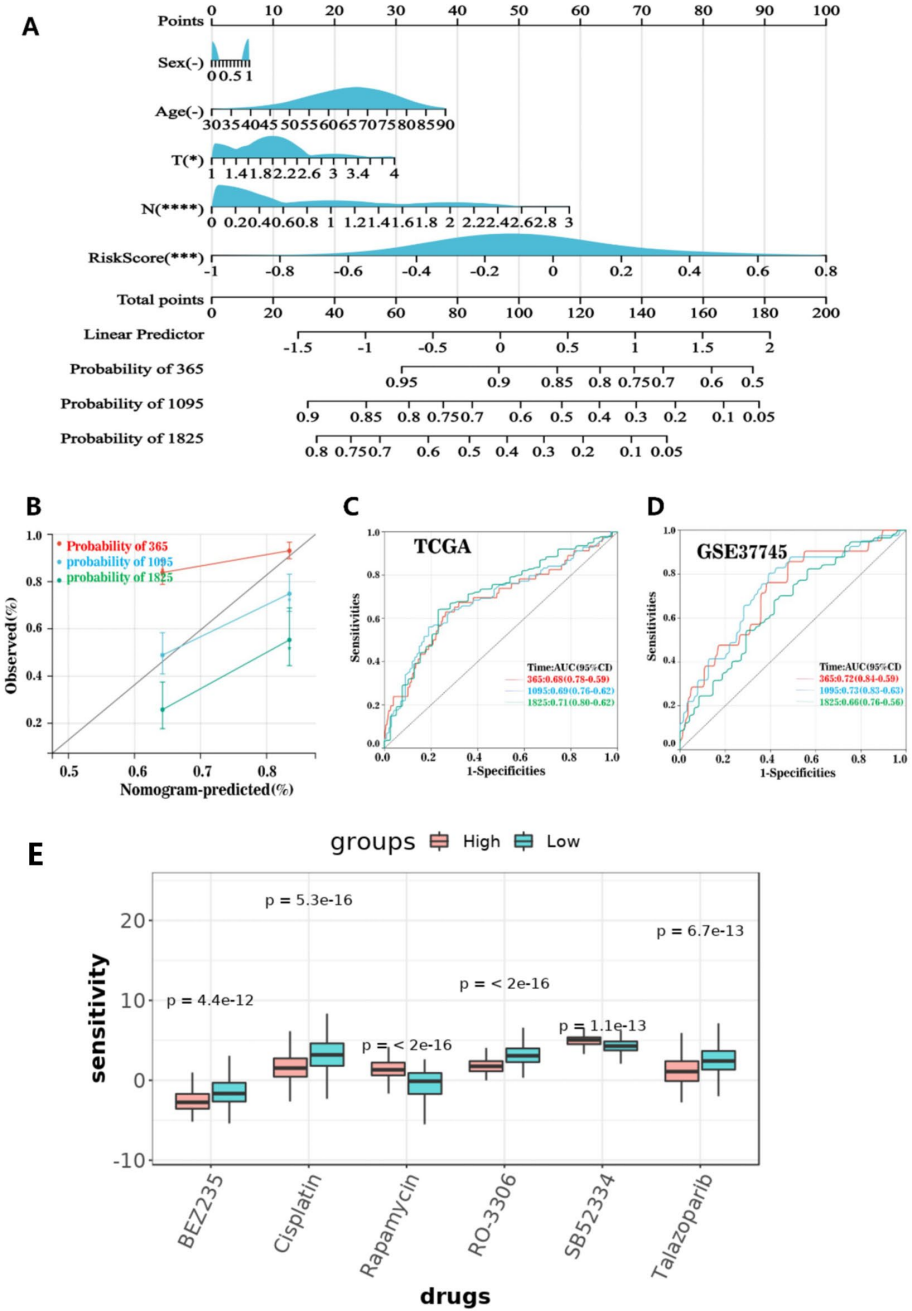
Creating a multifactor nomogram. (**A**) Nomograph used to estimate survival rates for patients with lung cancer at 1, 3, and 5 years. (**B**) Calibration curve of the nomogram. Kaplan–Meier OS curve and ROC curve of the (**C**) training and (**D**) verification sets. (**E**) Relationship between SFRG score and sensitivity to chemotherapy or targeted drugs in lung adenocarcinoma. BEZ235, cisplatin, RO-3306, talazoparib, rapamycin, and SB52334 were assessed. *P < 0.05; **P < 0.01; ***P < 0.001; ****P < 0.0001. OS, overall survival; ROC, receiver operating characteristic; SFRGs, SLC family-related genes.

We combined the survival time, survival status, and data for the five characteristics using the R “RMS” software package and used the Cox method to create a nomogram to forecast the survival status of patients with LUAD at 1, 3, and 5 years (Fig. 9A). The calibration curve for the patient nomogram is shown in Fig. 9B. Finally, the prognostic difference between the two groups was examined using the survfit function of the R “survival” package, which was also supported by the Gene Expression Omnibus (GEO) cohort research. The analysis of the receiver operating characteristic (ROC) curve verified that our prediction model was effective (Fig. 9C,D). We used the pRRophytic method to predict drug sensitivity in high-risk and low-risk cancer patients. The sensitivity to rapamycin and SB52334 was higher in high-risk patients than in low-risk patients, whereas sensitivity to BEZ235, cisplatin, RO-3306, and talazoparib was higher in low-risk patients than in high-risk patients (Fig. 9E).

### Q-PCR

**Figure 10:**
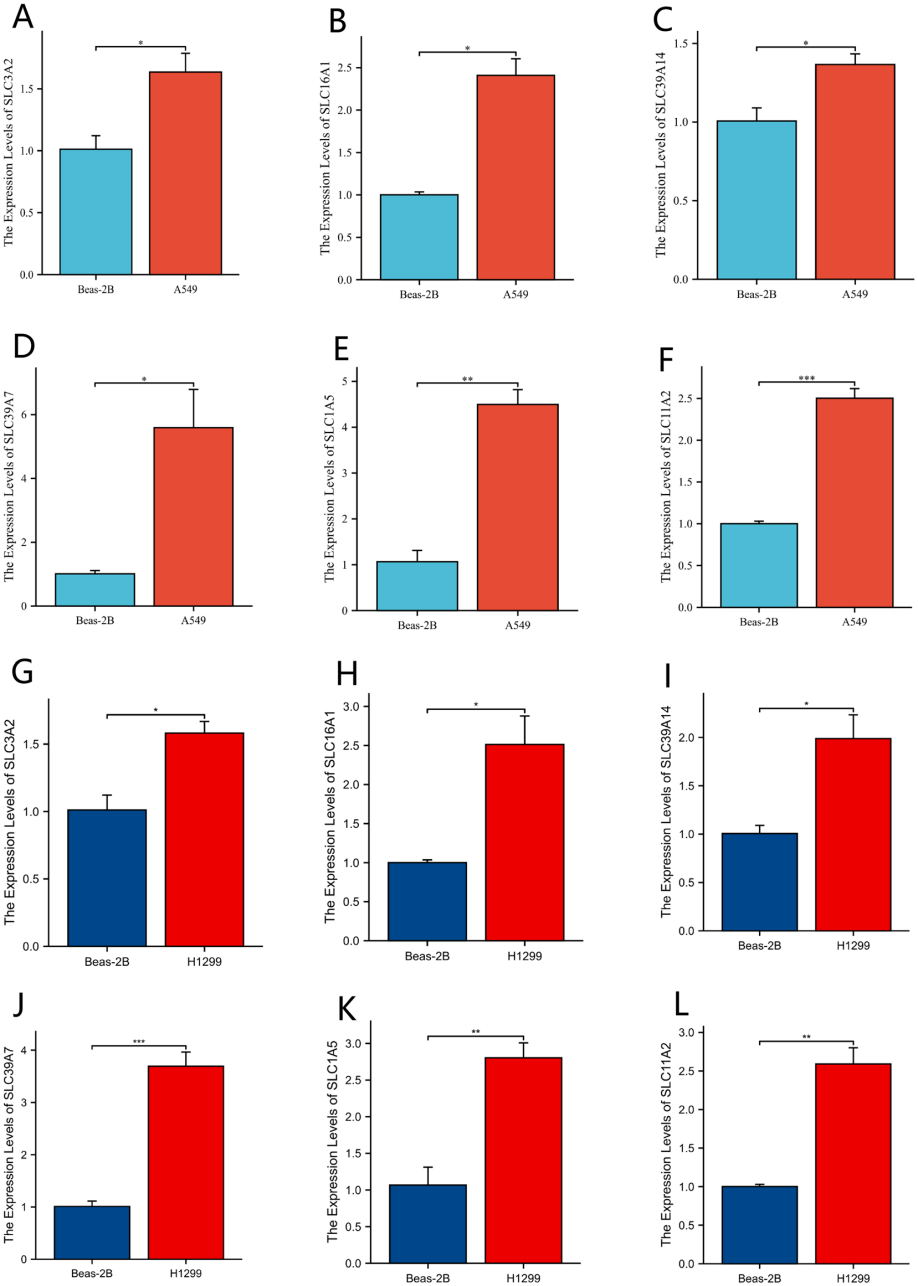
Q-PCR. (**A–L**) Gene expression difference between SLC3A2, SLC16A1, SLC39A14, SLC39A7, SLC1A5, and SLC11A2 in lung adenocarcinoma cell line A-549 and H1299, and normal lung epithelial cell line Beas-2B. *P < 0.05; **P < 0.01; ***P < 0.001.

The gene expression of SLC3A2, SLC16A1, SLC39A14, SLC39A7, SLC1A5, and SLC11A2 in the lung adenocarcinoma cell line A-549 and H1299 is higher than that in the normal lung epithelial cell line Beas-2B (Fig. 10A–L).

### Western blot

**Figure 11:**
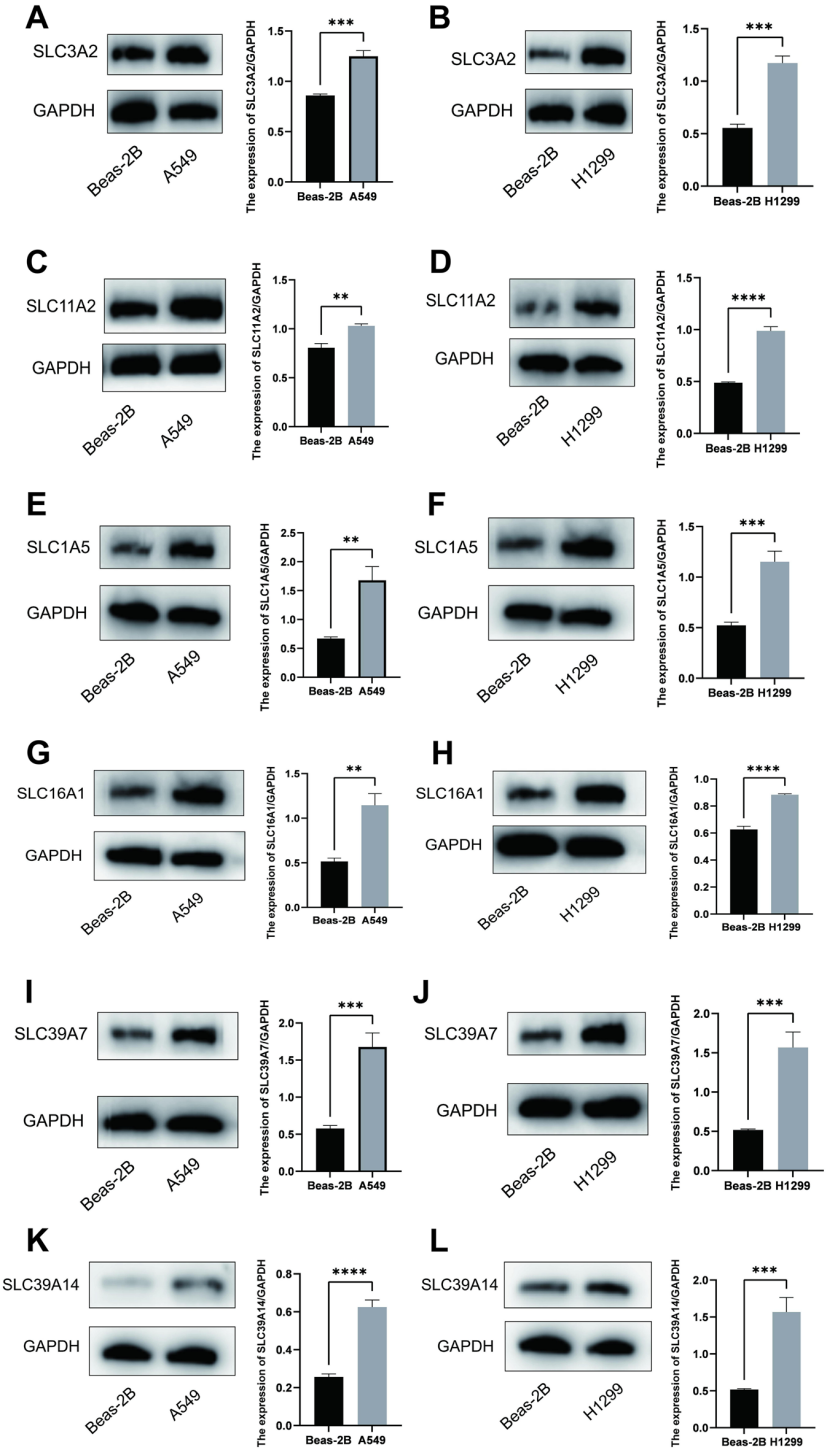
Western blot. (**A–L**) Protein expression difference between SLC3A2, SLC11A2, SLC1A5, SLC16A1, SLC39A7 and SLC39A14 in lung adenocarcinoma cell line A-549 and H1299, and normal lung epithelial cell line Beas-2B. **P < 0.01; ***P < 0.001; ****P < 0.0001.

The protein expression of SLC3A2, SLC11A2, SLC1A5, SLC16A1, SLC39A7, SLC39A14 in the lung adenocarcinoma cell line A-549 and H1299 is higher than that in the normal lung epithelial cell line Beas-2B (Fig. 11A–L).

## Discussion

Lung cancer exhibits a complicated pathogenesis and is characterized by a high degree of cell heterogeneity; it frequently invades the surrounding tissue and metastasizes. Therefore, lung cancer is often associated with resistance to a variety of targeted treatments^19^. Ferroptosis is a distinct form of iron-dependent programmed cell death that is characterized by the buildup of lipid peroxides and intracellular reactive oxygen species^20^. The use of the ferroptosis induction strategy to treat cancer is expanding^8^. Furthermore, the prerequisites for using ferroptosis-targeted medicines in clinical therapy are becoming increasingly established^21^. The SLC family proteins play crucial roles in fundamental biological processes and human disorders^22,23^, especially as transporters of metal ions, amino acids, and lipids in ferroptosis^24–26^. Therefore, the SFRGs involved in ferroptosis may be positive indicators of survival outcomes in patients with lung cancer. Based on the characteristics of genes associated with the SLC family in ferroptosis, we developed a model that can be used to determine the prognosis and immunotherapy response of patients with LUAD. Consensus clustering was used to categorize the SFRGs into two subgroups based on their expression. The C1 subtype outperformed the C2 subtype in terms of clinical survival rate, immunological rating, and immune cell infiltration. Furthermore, we chose three SFRGs (SLC16A1, SLC3A2, and SLC11A2) using the Lasso–Cox technique for regression analysis and developed a reliable risk model to divide patients with LUAD into high- and low-risk groups. The overall survival of patients can be accurately predicted using this technique and used as a standalone prognostic indicator for patients with LUAD.

The tumor microenvironment includes the surrounding blood vessels, immune cells, fibroblasts, bone marrow-derived inflammatory cells, various signaling molecules, and the extracellular matrix^27^. The onset and progression of ferroptosis alters the immunological milieu, which is crucial for immunotherapy^28^. In addition, ferroptosis produces lipids, and their interactions can influence the release of HMGB1, which controls tumor immunity^29,30^. Importantly, our approach may be helpful in analyzing variations in the immune microenvironment among various patients with adenocarcinoma. "Hot" tumors, also known as immune cell infiltrating tumors, are distinguished by a high infiltration of T lymphocytes surrounding and within the tumor^31^. Because T cell infiltration in C1 subtype patients is higher than in C2 subtype patients in our model, C1 subtype patients are more likely to have thermal tumors.

Our study has several significant limitations. First, it did not include samples from hospitalized patients, which could have improved the accuracy of this prediction model. Second, the fundamental experiments were insufficient to fully investigate our model. These issues will be addressed in further studies to ensure that the findings of our work are clinically translatable for future therapeutic applications.

## Conclusion

In this study, we developed a prediction model to forecast the outcomes of patients with LUAD and their immune features that may correlate with immune response by thoroughly analyzing SFRGs. Our study examined the significance of SFRGs from a variety of angles and established a benchmark for the future care of patients with LUAD.

## Methods

### Dataset

The Cancer Genome Atlas (TCGA; https://gdc.cancer.gov/)RNA sequencing (RNA-Seq) transcriptome data and associated clinicopathological data of 451 patients with LUAD were used to create the training set. The Gene Expression Omnibus (GEO; https://www.ncbi.nlm.nih.gov/geo/) provided a dataset of 105 patients with lung cancer that was used as the validation set (ID: GSE37745). We located SFRGs using the GeneCards (https://www.genecards.org/) and FerrDB (http://www.zhounan.org/ferrdb) databases.

### Consensus clustering

ConsensusClusterPlus was used to perform the cluster analysis, which involved resampling 80% of the samples 10 times and employing agglomerative partitioning around medoids (PAM) clustering with a 1-Pearson correlation distance.

### Identification of DEGs

The significance of each gene in the comparison and control groups was evaluated using the t-test function in R software. The screening conditions for screening were as follows: *P* < 0.05, FDR < 0.05, and |fold change| >1.5.

### Functional enrichment analysis

To compare the different signaling pathways and biological effects between the cluster1 and cluster2 cohorts, GO and KEGG^32–34^ studies were performed. To acquire the gene set enrichment findings of the gene set, we performed an enrichment analysis on the annotated genes using the R software package “clusterProfiler” (version 3.14.3).

### GSEA

To assess the relevant molecular processes and pathways, we divided the samples into two groups based on SFRG expression. Subsequently, we obtained a data subset from the Molecular Signatures Database (http://www.gsea-msigdb.org/gsea/downloads.jsp). The minimum and maximum number of gene sets was 5 and 5,000, respectively, and the resampling value was 1,000. GSEA software was used for the analysis (version 3.0, http://software.broadinstitute.org/gsea/index.jsp).

### Somatic mutation analysis

The gene mutations in 451 individuals with lung cancer were examined using the “maftools” function in R software. The results of the analysis are presented as waterfall diagrams.

### Immune landscape characteristics among patients in cluster1 and cluster2 subgroups

IOBR is an R software package used to study immunological tumor biology^35^. Based on our expression profile data, the score of infiltrating immune cells in the samples was calculated with IOBR, using ESTIMATE^36^, MCPcounter and TIMER^37,38^.

### Construction of a SFRG-related risk signature

We combined the data using the R software package “glmnet” and performed regression analysis using the Lasso–Cox technique. In addition, we calculated the best risk score cutoff value using the R software package “maxstat” and adjusted the minimum and maximum numbers of grouped samples to be larger than 25% and less than 75%, respectively. Therefore, the patients were divided into high- and low-risk groups.

### Creation and verification of multifactor nomogram

We used the R software package “RMS” to integrate data regarding survival time, survival state, and the five characteristics. Using the Cox technique, we created nomograms and assessed the prognostic importance of these characteristics in 425 samples. The total C-index, 95% confidence interval (CI), and *P*-value of the model were 0.66327, 0.60882–0.71772, and 4.1665, respectively.

### Drug sensitivity analysis

We examined the drug sensitivity of samples from the low- and high-risk groups using pRRophetics.

### Q-PCR

We compared the difference of SLC3A2, SLC16A1, SLC39A14, SLC39A7, SLC1A5, and SLC11A2 gene expression between normal pulmonary epithelial cell line Beas-2B and lung adenocarcinoma cell line A-549 by Q-PCR. SCL3A2 (5`-3`), F:AGCTGGAGTTTGTCTCAGGC, R:GGCCAATCTCATCCCCGTAG; SLC16A1 (5`-3`), F:TTTGGATTTGCCTTCGGGTG, R:TGAGCCGACCTAAAAGTGGT; SLC39A14 (5`-3`), F:AGAAGGTCATTGTGGGCTCG, R:AGTGAAGGAAGCACCGATGG; SLC39A7 (5`-3`), F:GGCTTAGACCTGCGTGTGTC, R:GCAAAGTCTCCGACCTCGTG; SLC1A5 (5`-3`), F:GGGTTTACTCTTTGCCCGCC, R:AAGCGGTAGGGGTTTTTGCG; SLC11A2 (5`-3`), F:TTGGGAAAACCAACGAGCAG, R:ATCCCCACTGCCCAAATGTA.

### Western blot

We cultured Beas-2B, A549, and H1299 cell lines in high glucose DMEM medium containing 10% fetal bovine serum (FBS). When extracting total proteins from cells, we used RIPA buffer (Beyotime Biotechnology, Shanghai, China) to obtain cell lysates on ice, while adding SDS-PAGE protein like buffer (Beyotime Biotechnology, Shanghai, China) to the lysates and heating them at 100 °C for 10 minutes. The total protein was injected into Bis Tris SDS/PAGE gel for electrophoresis, and transferred to polyvinylidene fluoride (PVDF) membrane for membrane transfer. After sealing with 5% bovine serum albumin (BSA) for 60 minutes, the membrane was incubated overnight with the primary antibody at 4 °C, and then exposed to the secondary antibody for 80 minutes. We processed the bands using the Enhanced Chemiluminescence (ECL) kit (Beyotime Biotechnology, Shanghai, China) and performed exposure analysis using an imaging system. Finally, use Image J software (National Institutes of Health (NIH), Bethesda, MD, USA) for protein quantification. All antibody information can be found in Supplementary Table 1.

### Survival Analysis

The prognostic differences between various sample groups were analyzed using the “survfit” function in the R software package, and the significance of these prognostic differences was determined using the log-rank test.

### Statistical analysis

All statistical analyses were carried out GraphPad Prism 9 (San Diego, CA, USA). All experiments were repeated at least three times, and the values are presented as the mean ± standard deviation (SD). Student’s t-test was used for comparisons between two groups. The statistical significance was set to *P* < 0.05, and the significance levels were expressed as *P < 0.05, **P < 0.01, ***P <0.001, and ****P < 0.0001. Non significant differences were represented as' ns'

### Supplementary Information

Supplementary Information 1.

Supplementary Information 2.

## Data Availability

The datasets for this study can be found in The Cancer Genome Atlas( https://gdc.cancer.gov/), the GeneCards (https://www.genecards.org/) and FerrDB (http://www.zhounan.org/ferrdb) databases. (GSE37745)datasets were downloaded from Gene Expression Omnibus(https://www.ncbi.nlm.nih.gov/geo/).
